# Effect of an exercise-based cardiac rehabilitation program “*Baduanjin* Eight-Silken-Movements with self-efficacy building” for heart failure (BESMILE-HF study): study protocol for a randomized controlled trial

**DOI:** 10.1186/s13063-018-2531-9

**Published:** 2018-03-01

**Authors:** Xiankun Chen, Wei Jiang, Xiaoli Lin, Cecilia Stålsby Lundborg, Zehuai Wen, Weihui Lu, Gaetano Marrone

**Affiliations:** 1 Department of Public Health Sciences, Global Health - Health Systems and Policy, Karolinska Institutet, 17177 Stockholm, Sweden; 2 Key Unit of Methodology in Clinical Research, Guangdong Provincial Hospital of Chinese Medicine, Guangzhou, 510120 China; 3 The Second Affiliated Hospital of Guangzhou University of Chinese Medicine, Guangzhou, 510405 China; 4 Department of Cardiology, Guangdong Provincial Hospital of Chinese Medicine, Guangzhou, 510120 China; 5 National Centre for Design Measurement and Evaluation in Clinical Research, Guangzhou University of Chinese Medicine, Guangzhou, 510405 China; 6 Heart Failure Center/Department of Cardiology, Guangdong Provincial Hospital of Chinese Medicine, Guangzhou, 510120 China

**Keywords:** Exercise-based cardiac rehabilitation, *Baduanjin*, Chronic heart failure, Exercise capacity, Quality of life

## Abstract

**Background:**

Exercise-based cardiac rehabilitation is a beneficial therapy for patients with chronic heart failure. The delivery of exercise-based cardiac rehabilitation should adopt an evidence-based approach, as well as be culturally appropriate and sensitive to individual needs and preferences. The *Baduanjin* Eight-Silken-Movements with Self-efficacy Building for Heart Failure (BESMILE-HF) program is the first to apply a traditional Chinese exercise, *Baduanjin*, as the core component in an exercise-based cardiac rehabilitation program. This trial aims to assess the efficacy, safety, and acceptability of the addition of the BESMILE-HF program to usual medications for patients with chronic heart failure.

**Methods/design:**

The BESMILE-HF study is a mixed-design study. It includes a two-group, parallel, randomized controlled trial with 200 chronic heart failure patients, as well as a qualitative component. Patients will be randomized into either an intervention group receiving the 12-week BESMILE-HF program plus usual medications, or a control group receiving only usual medications. The primary outcomes are peak oxygen consumption assessed using a cardiopulmonary exercise test, and disease-specific quality of life using the Minnesota Living with Heart Failure Questionnaire. The secondary outcomes are: exercise performance, exercise self-efficacy, general quality of life, dyspnea and fatigue, depression, cardiac function, prognostic and inflammatory indicator levels, hospitalization, use of medications, and major adverse cardiac events. Assessments will be carried out at baseline, and at the 4th week, 8th week, and 12th week. The qualitative component will include a semi-structure interview describing patients’ experiences with the intervention.

**Discussion:**

This study can provide evidence for how to deliver a contextually adapted exercise-based cardiac rehabilitation program with the potential to be scaled up throughout China.

**Trial registration:**

ClinicalTrials.gov, ID: NCT03180320. Registered on 2 June 2017.

**Electronic supplementary material:**

The online version of this article (10.1186/s13063-018-2531-9) contains supplementary material, which is available to authorized users.

## Background

Chronic heart failure (CHF) is present in approximately 1–2% of the adult population in high-income countries [1]. In China, 0.9% of the population has CHF, resulting in over four million sufferers [2]. Mortality rates for CHF are high, even for patients compliant with the best available pharmaceuticals and medical devices [3].

Exercise intolerance is a primary symptom among patients with CHF, and a strong determinant of prognosis as well as reduced quality of life. Therefore, exercise has been an accepted adjunct therapy for CHF [4], and has become a central component of cardiac rehabilitation. This is known as exercise-based cardiac rehabilitation (EBCR) which has been shown to improve CHF patients’ exercise capacities, as well as their quality of life [4, 5]. It has thus been widely recommended internationally [3, 6]. Despite its benefits, the uptake of EBCR remains suboptimal, primarily due to low availability, especially in low- and middle-income countries [7]. In addition to low availability, multiple barriers such as unaffordability as well as other barriers to exercise—often related to practical issues such as time, the practicality of travel to a rehabilitation center, or climate—prevent eligible patients from participating in EBCR programs or regular exercise [8]. Moreover, the hallmark symptoms (fatigue, dyspnea) and safety concerns frequently lead to decreased physical activity, and erode the self-confidence necessary to initiate and maintain regular exercise [9], especially for those with limited mobility.

Similar challenges of this poor EBCR compliance are also faced in China, where the key driver is low availability [10]. A recent national survey suggested that only 24% of the tertiary hospitals in China have EBCR services [11]. A local consensus document for EBCR is available in China [12], but the practice patterns for EBCR in hospitals, as well as other medical care units, are still rudimentary and not standardized. There are currently few cardiologists who regularly add EBCR to their daily clinical practice for CHF patients [11]. Unfortunately, the standardized EBCR delivery models in high-income countries are not contextually feasible in the Chinese setting due to the lack of rehabilitation facilities and trained professionals [11]. The delivery of EBCR in a specific setting should adopt an evidence-based approach, and more importantly, should be culturally appropriate and sensitive to individual needs and preferences [13].

Traditional Chinese exercise can play a role in the field of contextually adapted EBCR programs in China, and a recent systematic review supports its use for cardiovascular disease [14]. The physical effects of EBCR are related to its underlying exercise training intensity, and moderate-intensity aerobic exercise is recommended for an EBCR program for CHF [15]. Although traditional Chinese exercise is usually classified as low-intensity exercise for the healthy, it can be considered moderate- or even high-intensity aerobic exercise for patients with CHF, when considering their generally older age and low functional capacity [16]. In addition, traditional Chinese exercise is not only a physical, but also mental form of aerobic exercise which can improve perceived feelings such as quality of life [17]. This equipment-free exercise might be ideal for settings with limited resources [10], as well as for CHF patients, because it can be done at home, reducing barriers such as weather, transportation, and costs [8].

**Fig. 1:**
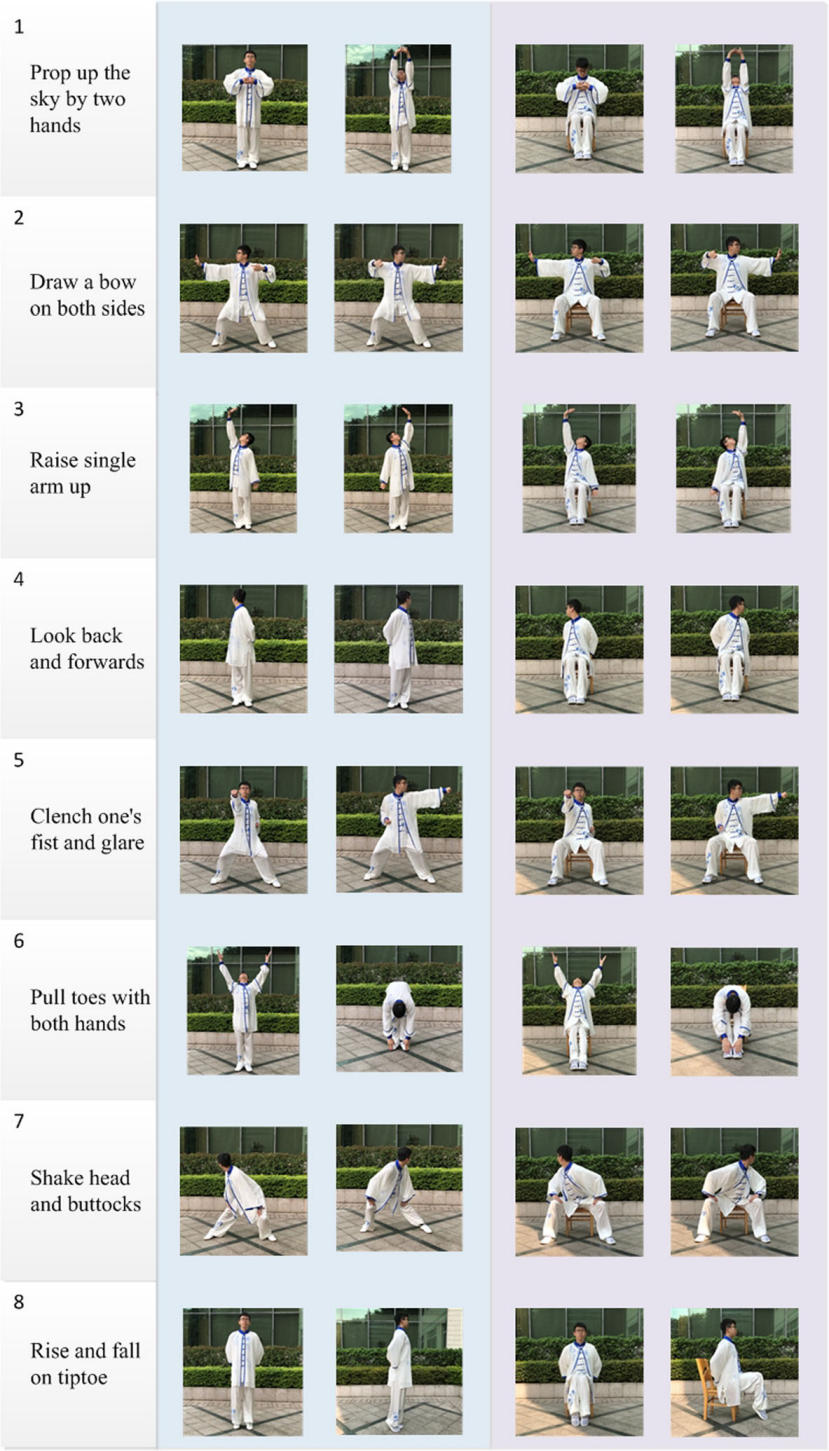
Baduanjin (standing and sitting forms) for the BESMILE-HF program

Two common traditional Chinese exercises are Tai Chi and *Baduanjin* (also called the “Eight-Silken-Movements” (see Fig. 1)). They are both commonly accepted as being beneficial to one’s health and have become community exercises throughout different regions in China. A multi-center randomized controlled trial (RCT) in the USA has shown that Tai Chi can significantly improve quality of life and motional status in patients with CHF, compared to usual care [18]. Although *Baduanjin* and Tai Chi share some common characteristics, *Baduanjin* is less demanding for older CHF patients because: it has fewer movements (eight versus 24 for Tai Chi); it requires shorter durations (15 min versus 30 min for Tai Chi); and it is possible to do while sitting (versus Thai Chi which only has a standing form). Moreover, *Baduanjin* has been shown to be effective for CHF-related medical issues such as fatigue, cardiorespiratory endurance, systolic and diastolic blood pressure, and resting heart rate [19]. Therefore, the integration of traditional *Baduanjin* in modern rehabilitation will provide CHF patients with a simple, inexpensive, and widely practicable EBCR delivery model which has the potential to be scaled up throughout China.

Accordingly, a new EBCR program, BESMILE-HF, has been developed at the Guangdong Provincial Hospital of Chinese Medicine (GPHCM)—a tertiary care hospital and one of the oldest and largest Chinese medicine hospital groups in China. **BESMILE-HF** is short for the ***B****aduanjin* **E**ight-**S**ilken-**M**ovements w**I**th Se**L**f-**E**fficacy building for **H**eart **F**ailure. *Baduanjin* has been applied for the first time as the core component in an EBCR program. The program also includes other components recommended in current guidelines such as education, evaluation, and consultancy [20], as well as a series of adherence strategies. Structured exercise trainings are not sufficient on their own: interventions must also include strategies to increase adherence and to maintain exercise compliance over time [21]. Self-efficacy (people’s belief and confidence in their capability to exercise across problematic situations) is reported to be the most dominant factor in the uptake and maintenance of exercise among the CHF population [22]. However, the value of the BESMILE-HF program has yet been proven, and, therefore, a clinical trial is needed.

This study (BESMILE-HF) aims to assess the efficacy, safety, and acceptability of the addition of the BESMILE-HF program to usual medications for CHF patients in China [23]. The primary hypothesis is that the BESMILE-HF program plus usual medications (as received by participants in the “intervention group”) compared with usual medications alone (as received by participants in the “control group”) can improve exercise capacity, measured as peak oxygen consumption (VO_2_) from a cardiopulmonary exercise test (CPX); also measured as quality of life based on total scores from the Minnesota Living with Heart Failure Questionnaire (MLHFQ) at 12 weeks (co-primary outcomes). Secondary objectives are to evaluate the effect of the BESMILE-HF program on: exercise performance, exercise self-efficacy, overall quality of life, hallmark symptoms such as dyspnea and fatigue, depression, cardiac function, levels of prognostic biomarkers/inflammatory indicators, hospitalization, use of medications and major adverse cardiac events. Other objectives are to explore the: (1) influence of potential factors on adherence to the BESMILE-HF program, (2) safety of the BESMILE-HF program, and (3) individual experiences and acceptability following the BESMILE-HF program.

## Methods/design

### Design

**Fig. 2:**
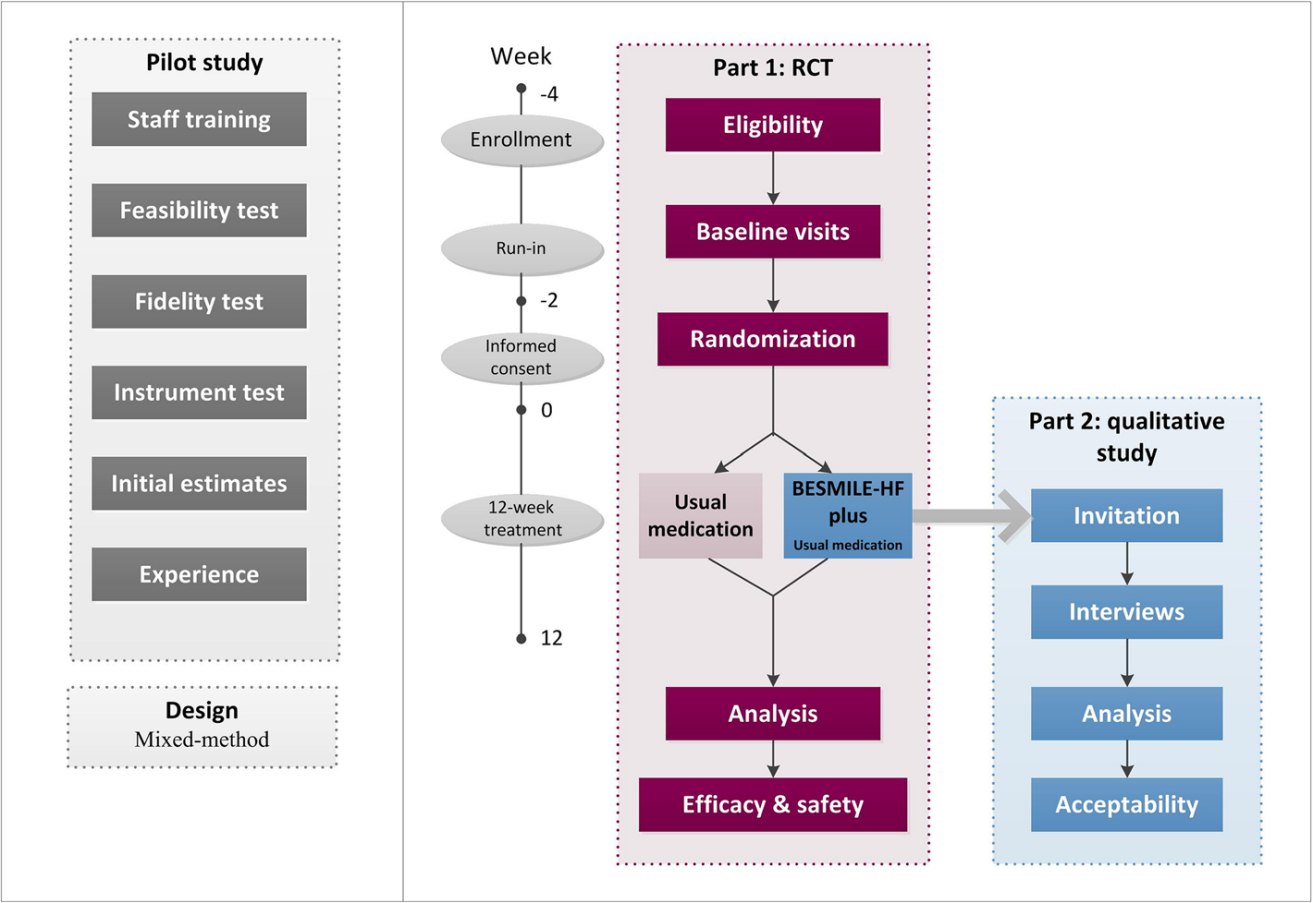
BESMILE-HF study evaluation processes. RCT randomized controlled trial

**Fig. 3:**
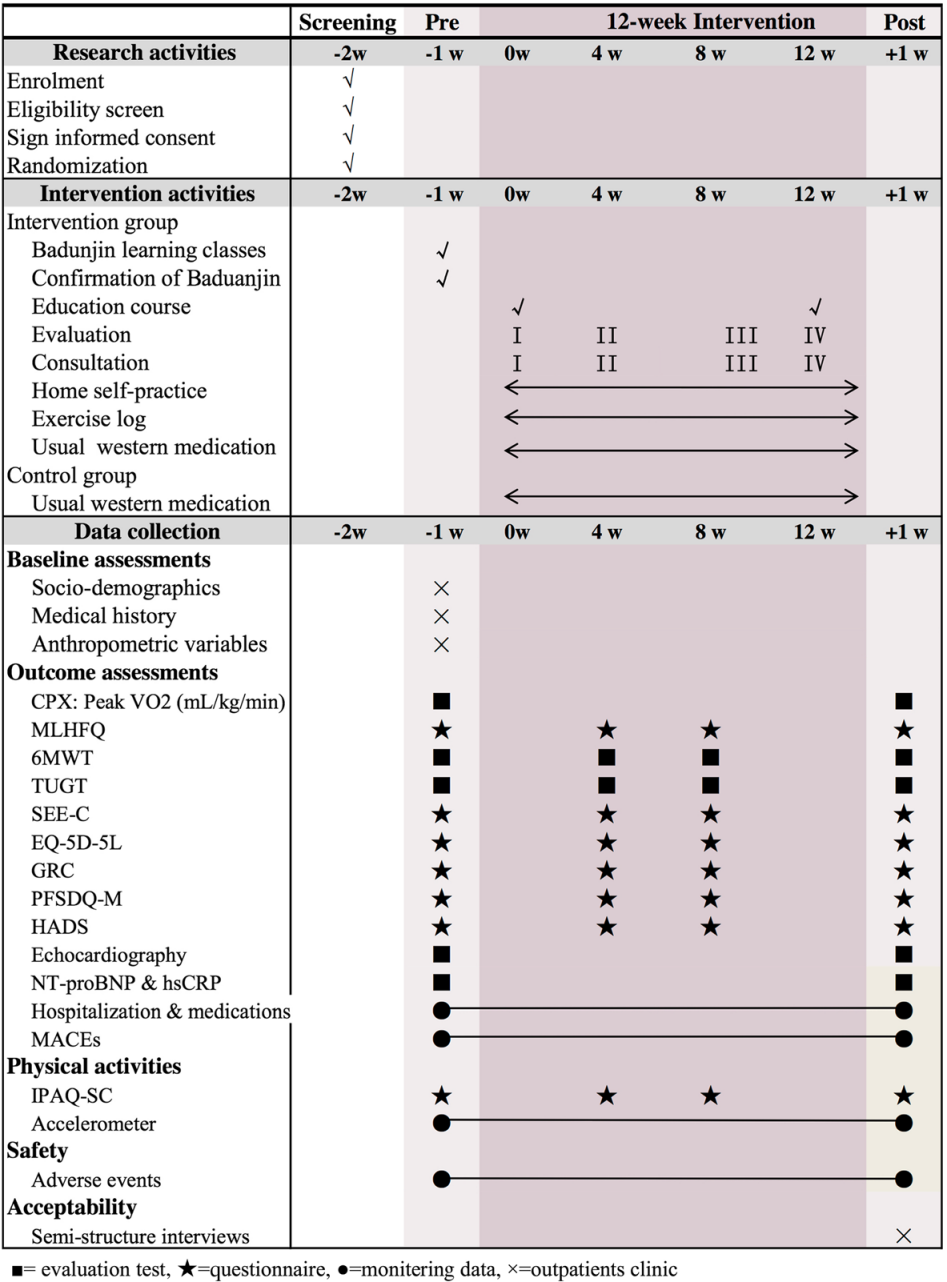
BESMILE-HF study schematic diagram. w week, CPX cardiopulmonary exercise test, VO_2_ oxygen consumption, MLHFQ Minnesota Living with Heart Failure Questionnaire, 6MWT 6-min walking test, TUGT Timed up-and-go test, SEE-C Self-Efficacy for Exercise – Chinese, GRC Global Rating of Change, PFSDQ-M Modified Pulmonary Functional Status and Dyspnea Questionnaire, HADS Hospital Anxiety and Depression Scale, NT-proBNP N-terminal B-type natriuretic peptide, hsCRP high-sensitivity C-reactive protein, MACEs major adverse cardiac events, IPAQ-SC Chinese version of the International Physical Activity Questionnaire short-form

The BESMILE-HF study is registered on ClinicalTrial.gov (NCT03180320) and consists of two main parts. A brief flowchart of the entire study is shown in Fig. 2, and the schedule of events is provided in Fig. 3.

1. Part 1: this will be a two-group, parallel, RCT to evaluate the efficacy and safety of the BESMILE-HF program for CHF patients. Eligible participants will be randomized to either an intervention group receiving a 12-week BESMILE-HF program plus usual medications, or a control group receiving only the usual medications. This protocol includes the recommended elements elaborated upon in the Standard Protocol Items: Recommendations for Interventional Trials (SPIRIT) Checklist (Additional file 1) [24]
2. Part 2: this will be a qualitative study using semi-structured interviews to better understand the individual experience and acceptability of participants enrolled in the BESMILE-HF program
3. A pilot mixed-design study will be conducted before the full-scale trial. It will assess the main uncertainties that have been identified in the development work, and will focus on the following metrics: (1) training research staff, (2) testing the feasibility of trial processes (such as rate of recruitment, eligibility, retention, attrition, and follow-up response), (3) testing the fidelity of intervention, (4) testing data collection instruments and procedures, (5) attaining initial estimates of the effects of primary outcomes, and (6) exploring the participation experience

### Setting and participants

The BESMILE-HF study will be conducted in Guangzhou in Southeastern China. Guangzhou is China’s third largest city and has a population of 13,501,100 [25]. Patients will be recruited from cardiology outpatient clinics at each of the four GPHCM branch hospitals in the different urban districts of Guangzhou. To identify potential participants for enrolment, we will use: (1) on-site screening of patients at clinic visits; (2) regular screening of potential participants using electronic medical records; and (3) referrals from physicians and other providers. The complete list of inclusion and exclusion criteria is provided in Table 1.

**Table 1:** BESMILE-HF study inclusion and exclusion criteria

Inclusion criteria
1. Aged 18 years or above
2. Diagnosed with chronic heart failure
3. Clinically stable, defined as symptoms/signs that have remained generally unchanged for ≥ 1 month
4. New York Heart Association functional classification of II or III
5. Informed consent provided
Exclusion criteria
1. Patients who have contraindications to exercise testing: early phase after acute coronary syndrome (up to 6 weeks); life-threatening cardiac arrhythmias; acute heart failure (during the initial period of hemodynamic instability); uncontrolled hypertension (systolic blood pressure > 200 mmHg and/or diastolic blood pressure > 110 mmHg); advanced atrioventricular block; acute myocarditis and pericarditis; moderate to severe aortic/mitral stenosis; severe aortic/mitral regurgitation; severe hypertrophic obstructive cardiomyopathy; acute systemic illness; or intracardiac thrombus.
2. Patients who have contraindications to exercise training: progressive worsening of exercise tolerance or dyspnea at rest over the previous week; significant ischemia during low-intensity exercise (< 2 metabolic equivalents, < 50 W); uncontrolled diabetes; recent embolism; thrombophlebitis; or new-onset atrial fibrillation/atrial flutter
3. Patients who have serious acute or chronic disease affecting major organs, or mental disorders
4. History of cardiac surgery, cardiac resynchronization therapy, intracardiac defibrillation, or implantation of combined device within the previous 3 months
5. History of cardiac arrest within 1 year
6. History of peripartum cardiomyopathy, hyperthyroid heart disease, primary pulmonary hypertension
7. Inability to perform a bicycle stress test
8. Severe cognitive dysfunction precluding understanding of exercise concepts
9. Current participation in either Baduanjin or a conventional cardiac rehabilitation program
10. Participation in a concurrent trial

### Randomization, allocation concealment and blinding

After informed consent is signed, patients will be randomized into either an intervention group receiving a 12-week BESMILE-HF program plus usual medications, or a control group receiving only usual medications. A block randomization sequence will be generated by SAS 9.2 (SAS Institute Inc., Cary, NC, USA) in a 1:1 ratio. Treatment assignments will be conducted using a web-based allocation system. Given the nature of the intervention, it is impossible to blind the patients and personnel involved in conducting the programs. Outcome assessors, laboratory technicians, data managers, and statisticians will be unaware of the treatment allocations.

### Intervention and control

The treatment delivery processes for both the intervention and control groups are outlined in Fig. 3.

### Control group

Patients in the control group will receive only the usual medications according to national guidelines. This is because patients typically do not receive EBCR in this type of setting [11].

### Intervention group

**Fig. 4:**
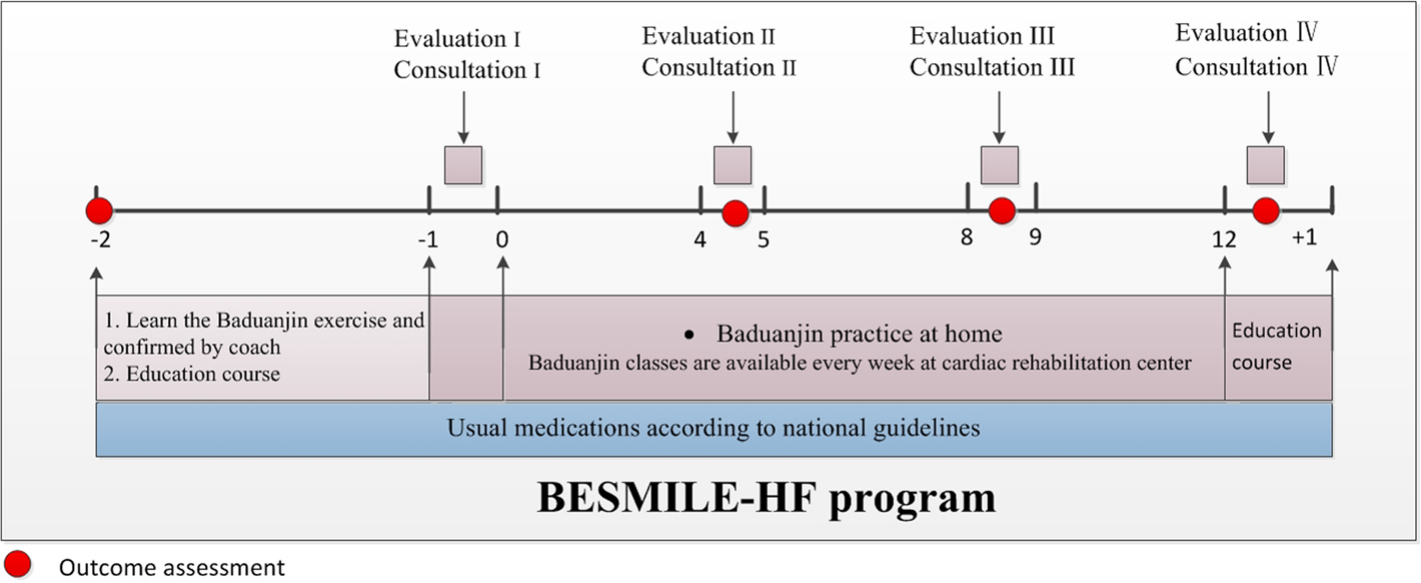
BESMILE-HF program procedures

Patients in the intervention group will receive usual medications according to national guidelines plus a BESMILE-HF program which will be conducted by a cardiac rehabilitation team consisting of cardiologists, cardiology nurses, coaches, physiotherapists, and research assistants. Procedures and activities of the BESMILE-HF program are listed in Fig. 4 and include: (1) *Baduanjin*, (2) Evaluations of exercise capacity and clinical conditions, (3) Consultations on exercise prescription and disease management, (4) education covering topics related to exercise for CHF and exercise, and (5) a series of adherence strategies derived from Bandura’s social cognitive theory [26] and designed to build exercise self-efficacy. Self-efficacy is centered on four core elements: “performance accomplishment”, “role modeling,” “positive feedback,” and “recognition of problems and problem-solving.” Specific adherence strategies for each of these elements of self-efficacy will be adopted and delivered as adjuncts to the *Baduanjin* within the BESMILE-HF program throughout the study period. Additional BESMILE-HF program details are listed in Additional file 2.

#### BESMILE-HF program pre-phase: 2 weeks before the start of exercise

1. Baduanjin training class
  - We will employ two professional coaches with at least 5 years of Baduanjin teaching experience to teach and guide participants’ training
  - Patients will be required to learn Baduanjin until they master it, as will be confirmed by the professional coaches
2. Educational course covering following topics
  - Basic knowledge of chronic heart failure
  - Basic knowledge of exercise-based cardiac rehabilitation
3. Initial evaluation of patients’ exercise capacity (week − 1 to week 0):
  - Evaluation is conducted by cardiologists and physiotherapists
  - Evaluation is based on reviewing medical history, cardiopulmonary exercise test results, and performance of Baduanjin (heart rate and oxygen consumption will be recorded while performing Baduanjin)
  - Evaluation results will guide the goal-setting process during consultation
4. An initial consultation lasting 20–30 min will be conducted by cardiologists and nurses (week − 1 to week 0):
  - Development of exercise prescription: cardiologists and nurses will work collaboratively with patients to set goals, guided by the initial evaluation and the following rules:
    In general, the recommended exercise volume is to do *Baduanjin* for 30 min per day, 5 days per week, for a total of 150 min per week [15, 27]
    Patients should be encouraged to progressively increase exercise duration, as tolerated, until they are able to tolerate one 30-min session [28]
    Patients with a baseline exercise capacity < 3 metabolic equivalents (METs) should be encouraged to start with the sitting-form *Baduanjin*, and patients with exercise capacity > 3 METs can start with the standing-form *Baduanjin* [29]
  - Medication management, recognition and problem-solving of symptoms and signs during exercise

#### BESMILE-HF program exercise phase: a 12-week intervention period

1. Baduanjin exercise:
  - Home exercise: participants will be encouraged to do Baduanjin at home with the instruction of a picture-based brochure
  - Alternatively, for those who would like to practice in a classroom setting, they will be able to attend a coach-guided class held at the rehabilitation center after making appointments with the research assistants. In the class, professional coaches will demonstrate exercise techniques, evaluate the participants’ performance, and provide personal feedback
  - Baduanjin classes will be available at a variety of times, including weekdays, weekends, and evening sessions, throughout the study period
  - Exercise logs will be designed to record each exercise session daily, including the duration in both minutes and frequency
2. Three evaluations, at the 4th week, 8th week, and 12th week
  - Re-assessment of exercise capacity and performance of Baduanjin
  - Clinical conditions and relevant physical examinations
3. Three consultations, at the 4th week, 8th week, and 12th week:
  - Difficulties in goal accomplishment and revision of exercise prescription
  - Medication management, recognition and problem-solving of symptoms and signs during exercise
4. Patients will be required to attend educational courses at the 12th week which cover the following topics:
  - Long-term maintenance of exercise
  - Disease management in daily life

### Outcomes measures

#### Primary outcomes

Two co-primary outcomes are the change in exercise capacity measured as peak VO_2_ (mL/kg/min) from CPX and disease-specific quality of life measured as the MLHFQ total scores between baseline and 12 weeks. Co-primary endpoints are chosen so that a comprehensive picture of the treatment effect can be obtained [30]. The objectives of cardiac rehabilitation are to positively influence disease progression and prognosis; and to improve patients’ overall quality of life [28]. Exercise capacity and quality of life are two different domains of interest in rehabilitation research [30]. Peak VO_2_ is the “gold standard” for assessing exercise capacity, and is an important predictor of prognosis in CHF patients [31]. MLHFQ is a validated and commonly employed disease-specific quality of life instrument in CHF which covers physical, psychological, and socioeconomic dimensions [32].

#### Secondary outcomes

- Exercise performance using the 6-min walking test (6MWT) and the Timed up-and-go test (TUGT)
- Exercise self-efficacy using the Self-Efficacy for Exercise-Chinese scale (SEE-C)
- Generic quality of life using the EuroQoL five-dimension, five-level (EQ-5D-5 L) questionnaire and the Global Rating of Change (GRC)
- Hallmark symptoms of dyspnea and fatigue using the Modified Pulmonary Functional Status and the Dyspnea Questionnaire (PFSDQ-M)
- Depression status using the Hospital Anxiety and Depression Scale (HADS)
- Cardiac function using echocardiogram and electrocardiograph (ECG)
- Levels of prognostic biomarkers (N-terminal B-type natriuretic peptide, NT-proBNP) and inflammatory indicators (high-sensitivity C-reactive protein, hsCRP)
- Hospitalization and use of medications
- Major adverse cardiac events (MACEs)

### Data collection

The BESMILE-HF study data collection process is outlined in Fig. 3.

### Baseline data

The following baseline data will be collected by questionnaires and through medical chart review: (1) socio-demographics, (2) medical history, and (3) anthropometric variables.

### Outcome assessments

Each participant will be asked to attend an in-person assessment appointment at the GPHCM Heart Failure Center at four time points: baseline, 4th week, 8th week, and 12th week. During each assessment, participants will be asked to complete specified physiological tests and self-reported questionnaires. All tests will be administered by one of two trained independent assessors.

### Co-primary outcome measurements

#### Peak VO_2_

The CPX test will be performed at baseline and at the 12th week according to the guidelines [31]. Technicians and physicians performing the tests will be blinded to the study group. Participants will perform a symptom-limited exercise test using a bicycle ramp protocol (10 W/min) to determine peak VO_2_. Testing will be done on an electronically calibrated upright bicycle, with expired gas analysis under continuous electrocardiographic monitoring. Blood pressure will be taken at 3-min intervals and just prior to starting/stopping exercise. Participants will be encouraged to exercise until exhaustion. Perceived exertion will be measured using the 6–20 Borg Scale. Respiratory gas analysis will be performed on a breath-by-breath basis using a metabolic cart (Cardiovit CS-200 Touch, SCHILLER, Baar, Switzerland). The peak VO_2_ will be defined as the highest value for or the plateau of oxygen uptake.

#### Disease-specific quality of life

The disease-specific quality of life will be self-assessed by the MLHFQ at baseline, the 4th week, 8th week, and 12th week. This validation instrument will consist of 21 items rated on 6-point Likert scales, representing different degrees of impact of CHF on quality of life, from 0 (none) to 5 (very much). It will provide a total score (range 0–105, with a lower number denoting better quality of life), as well as scores for two dimensions, physical (eight items, range 0–40) and emotional (five items, range 0–25). Prior studies have reported that a score of 7 indicates some degree of impaired quality of life, and that an improvement of 5 points represents a clinically significant change [33]. A validated Chinese version of the MLHFQ will be employed in this trial [32].

### Secondary outcome measurements

#### Exercise capacity

The 6MWT will be performed at baseline, 4th week, 8th week, and 12th week. The walking distance in meters within 6 min will be measured according to the American Thoracic Society guidelines [34]. The test will be done at least 2 h before the CPX test. An assistant blinded to the study group will read standardized, scripted instructions to each patient. Patients will be instructed to walk as far as possible and allowed to stop as often as they need if they are fatigued. The 6MWT is one of the most common instruments for measuring changes in exercise tolerance following exercise-based interventions in patients with CHF [35].

The TUGT will be performed at baseline, the 4th week, 8th week, and 12th week. It appears to be a reliable and valid functional measurement in patients with CHF, and is frequently used in rehabilitation settings as a measure of balance and mobility [36]. Patients are required to stand up from a 45-cm-high chair, walk 3 m at a comfortable pace, turn 180°, return to the starting point, and sit again. This test will be timed with the use of a standard stopwatch by a research assistant blinded to the treatment arm.

#### Exercise self-efficacy

Exercise self-efficacy will be self-assessed by the SEE-C at baseline, the 4th week, 8th week, and 12th week. The participants will be instructed to listen to nine different situations, and then to choose an option from 0 (not confident) to 10 (very confident) that represents their perception of confidence regarding engaging in regular exercise. The scale will be scored by summing the numerical ratings for each response and dividing the total by the number of non-missing responses. The mean scores for the self-efficacy of exercise will range from 0 to 10, with the higher scores representing greater exercise self-efficacy. A validated Chinese version of the SEE-C will be employed in this trial [37].

#### Generic quality of life

Generic quality of life will be self-assessed by the EQ-5D-5 L questionnaire at baseline, the 4th week, 8th week, and 12th week. This questionnaire has been used previously to measure health status in patients with heart failure [38, 39]. The EQ-5D-5 L questionnaire measures five items (mobility, self-care, usual activities, pain/discomfort, and anxiety/depression), with each item including five levels: no problems, some/moderate problems, and severe/extreme problems. In addition to the five health-state items, the EQ-5D also contains a Visual Analog Scale, a graph representation similar to a thermometer that ranges from 0 (worst imaginable health state) to 100 (best imaginable health state). It has been used previously to measure health status in patients with heart failure. A validated Chinese version of EQ-5D-5 L will be employed in this trial [40].

Subjective health will be self-assessed by the GRC at baseline, the 4th week, 8th week, and 12th week. The GRC will use a single item that compares current subjective health with previous subjective health at baseline. The response scale will range from − 7 (much worse) over 0 (no change) to 7 (much better) [41].

#### Hallmark symptoms of dyspnea and fatigue

Dyspnea and fatigue will be self-assessed by the PFSDQ-M at the baseline, 4th week, 8th week, and 12th week. The PFSDQ-M is comprised of 40 items divided into three components: dyspnea, fatigue, and activity. The activity domain evaluates changes in ten activities beginning at the point the patient first developed CHF: brushing/combing hair; putting on a shirt; washing hair; showering; raising arms overhead; preparing a snack; walking 10 feet; walking on inclines; walking on bumpy terrain; and climbing three stairs. The dyspnea component evaluates dyspnea for frequency during the preceding month; intensity of dyspnea on most days, the present day, and with usual activity levels (rated on a scale from 0 to 10); and intensity of dyspnea when the above-mentioned ten activities are performed (rated on a scale from 0 to 10). The fatigue component evaluates fatigue for the same items as the dyspnea component. Higher scores indicate more severe symptoms. The PFSDQ-M is validated and is reliable in patients with CHF. A validated Chinese version will be employed in this trial [42].

#### Depression status

The HADS will be self-administered at the baseline, 4th week, 8th week, and 12th week to measure the presence of depression and anxiety. It has been shown to be a valid and reliable measure of the severity of emotional disorders and is used in general hospital practice. Participants will be instructed to choose one response from the given answers that best describe their current feelings. The HADS is a 14-item self-report screening scale originally developed to indicate the possible presence of anxiety and depressive states in the setting of a medical outpatient clinic.

#### Cardiac function

Echocardiography: left ventricular ejection fraction (LVEF) will be assessed by echocardiography at baseline and at the 12th week. Each participant will undergo an initial resting three-dimensional, multi-view echocardiogram before the exercise program is initiated. Another echocardiogram will be taken upon completion of the program. LVEF will then be computed by dividing the stroke volume by the end-diastolic volume. In addition, other parameters related to diastolic/systolic/valvular status will also be collected.

A 12-lead ECG and Holter 24-h ECG will be conducted at baseline and at the 12th week. Data will be collected on a variety of parameters reflecting cardiac function such as rhythm, heart rate, duration of PQ interval (ms), QRS interval (ms), QT interval (ms), and QTc interval (ms), presence of second or third degree atrioventricular block, bundle-branch configuration, and tachycardia with narrow or wide QRS or delta waves. In addition, subjects’ autonomic nervous system function will be assessed using heart rate variability.

#### Levels of prognostic biomarkers and inflammatory indicators

Blood samples will be collected at baseline and 12 weeks to measure the levels of prognostic biomarkers (NT-proBNP) and inflammatory mediators (hsCRP). NT-proBNP and hsCRP will be analyzed by the GPHCM laboratory. The NT-proBNP is correlated with risk for all-cause, cardiac, and pump-failure mortality, and hsCRP is elevated in CHF as the disease progresses [6].

#### Hospitalization-related outcomes and medication usage

Hospitalization-related outcomes: all instances of hospitalization will be recorded throughout the study period and made accessible to an independent adjudication panel consisting of three experienced cardiologists. They will ascertain whether or not the reported events are heart failure-related.

Medication usage: usual medical management as well as concomitant medications will be documented in detail by cardiologists. In addition, concomitant medications must be recorded in participants’ exercise logs. Medical records, interview data, and automated utilization data will also be used to document patients’ use of healthcare throughout the study period.

#### MACEs

The MACEs are the most commonly used composite endpoints in cardiovascular research, and thus will be recorded throughout the study period [43]. MACE components in our study include events of death, myocardial infarction, stent thrombosis, percutaneous coronary interventions, coronary artery bypass graft surgery, and stroke.

### Physical activity level

The physical activity levels of all participants will be measured in two ways: (1) step counts, and (2) self-reported questionnaires. The step count will be measured using an accelerometer (Garmin Vivofit) that we will provide to each participant, both in the intervention and control groups. Participants will be required to wear the step count all day throughout the study period. Patients will return the step counts, and the ensuing data will be downloaded by research assistants. In addition, physical activity will be self-reported using the validated Chinese version of the International Physical Activity Questionnaire short-form [44] at the baseline, 4th week, 8th week, and 12th week.

### Safety

An independent Data and Safety Monitoring Committee will evaluate the progress of the study and assess the safety data, as requested during the study. Adverse events (AEs) will be defined as any undesirable experience participants endure during the trial period, regardless of whether or not it is associated with the intervention. Participants will be instructed to report AEs to the research team at any time, and a research nurse will monitor participants for potential occurrences of AE. All details of AEs, such as time of occurrence, severity, management, and causality to the intervention will be recorded on Case Report Forms (CRFs). All AEs will be followed up from the date they are brought to the investigator’s attention until resolution. Severe AEs must be reported to both the GPHCM Data and Safety Monitoring Committee and the GPHCM Ethics Committee within 24 h. Severe AEs will be defined according to the International Conference on Harmonization guidelines [45]: any adverse event will be regarded as serious if it results in death, is life-threatening, requires hospitalization or prolongation of existing hospitalization, or results in persistent or significant disability or incapacity.

### Qualitative data

Participants who are randomized to the RCT intervention group will be selected for the qualitative study. A purposive sampling strategy will be used based on maximum variation principals regarding age, gender, severity of CHF, education level, working status, whether having grandchildren, and the preliminary results of the pilot study. Participants will be recruited until saturation is reached, and the sample size is expected to be between 15 and 20. Data saturation is defined as no new information having been obtained from further interview. All participants will provide additional informed consent before taking part in this qualitative study.

Two female research assistants will conduct the interviews: one a nurse in the department of cardiology, and a resident physician with a background in cardiology. Both assistants will receive education and training in conducting interviews. They will not be part of the BESMILE-HF research team; however, the BESMILE-HF study research staff will inform them of the purposes of this qualitative study.

The semi-interviews will be conducted in a quiet room in the GPHCM or at patients’ homes, depending on patient preference. The duration of the interviews will range from 1 to 1.5 h, and all will be recorded and transcribed verbatim. An interview guide will be developed based on a review of the relevant academic literature, and will be piloted before use. During the interviews, participants will be encouraged to speak freely about their experiences with and perspectives on the entire BESMILE-HF program, motivating and inhibiting influences of the program, and suggestions for improvement. Subsequently, they will primarily be asked about their preferences, attitudes, use, abilities, and challenges regarding *Baduanjin*. All questions will be open-ended, and follow-up questions will be used to gain a deeper understanding of areas that appear essential for each individual.

### Statistical methods

#### Sample size calculation

Sample size calculation will be based on the co-primary outcomes of the RCT. A sample of 78 patients per group will provide 80% power to detect the difference in both peak VO_2_ = 1.5 (mL/kg/min) and MLHFQ total score = 10 points between groups at a two-tailed significance level of 5%. This accounts for the standard deviation of peak VO_2_ = 3 mL/kg/min and MLHFQ score = 20 points and meets clinically important difference (peak VO_2_ = 1 mL/kg/min [46] and MLHFQ score = 5 points [33]). This would require 156 participants, inflated to 200 to account for the loss to follow-up of approximately 20% of participants. Because each primary endpoint can characterize a clinically meaningful benefit of the intervention on its own, we will use the “or decision rule,” meaning that the study will be regarded as successful if one of the two primary endpoints improves significantly in the intervention group.

### Quantitative data analysis

Statistical analysis will be performed in a blinded manner by qualified statisticians using PASW Statistics 18.0 (IBM SPSS Inc., Armonk, New York, USA), SAS 9.2 (SAS Institute Inc., Cary, USA), and Stata (Version 13.0, StataCorp). The level of significance will be set at *p* < 0.05. Baseline variables will be reported by groups, descriptively. For the outcome variables, continuous data will be summarized as mean and standard deviation or range, or as median and interquartile range, and categorical data as counts and percentages. The primary analyses for primary and secondary outcome variables will be based on the intention-to-treat population approach, using data collected at baseline and at the 12th week. Differences in terms of outcomes between groups will be compared using an independent *t* test/Mann-Whitney *U* test, as appropriate. For the two co-primary outcomes, the significance level will be Bonferroni-corrected (*α* = 0.05/2 = 0.025). The effect of intervention on outcomes will also be analyzed using multivariate statistical models, with adjustments for baseline score of the outcome variable (where applicable), as well as gender, age, co-morbidities and other variables that are of clinical importance. In addition, multiple imputation methods will be used as a sensitivity analysis to address the issue of missing data. Subgroup analysis will be performed according to gender, cardiac functional classification, left ventricular ejection fraction classifications, and other variables of clinical importance. The secondary analyses for outcome variables will be undertaken as repeated measure analysis and mixed-effects models using all assessment points (4, 8 and 12 weeks). Sensitivity analysis will be performed, including, but not limited to, the analysis for outcome variables using a per-protocol population. Safety will be evaluated by tabulations of AEs, and will be presented with descriptive statistics for each group. A chi-square test or a Fisher’s exact test will be used to compare the frequency difference in AEs between the two groups. To explore potential factors which might influence adherence, a logistic regression model and a linear mixed-effects model will be used and adjusted for potential confounders. Adherence-related data will be taken from exercise log records.

### Qualitative data analysis

For qualitative data, content analysis based on an inductive approach will be conducted and assisted by NVivo v11. Categories and themes will be identified inductively. Analysis will be conducted by three researchers (RA, RB, RC) in three steps. In the first step, RA and RB have independently created two coding structures by reading three to four of the transcribed interviews and extracting meaningful units from the text; RA and RB will then meet to compare the coding structures, discuss differences and agree on a final version. Preliminary findings will be discussed with RC, and feedback will be incorporated into the coding structure. RA and RB will then apply the final coding structure to all transcripts. In the second step, categories will be created from the codes. The structure of the categories will be discussed among the research team. In the final step, categories with similar content will be grouped together into themes. To ensure the reliability of the findings: (1) the interviews will be read repeatedly to obtain an overall understanding of the project, and there will also be a constant comparison between the parts of the analysis and the full text of the interview and (2) awareness of preconceptions will be emphasized throughout the study.

### Trial management and quality control

The management structure is comprised of principle investigators (PIs) including Dr. Weihui Lu, Dr. Wei Jiang, and Dr. Gaetano Marrone, a Trial Management Group, a Data Monitoring Committee, and a Trial Steering Committee.

The Trial Management Group is responsible for the day-to-day delivery and for conducting the trial. It will be comprised of a project manager as well as a cardiac rehabilitation team and investigators from the GPHCM. The Trial Management Group will meet weekly to discuss trial progress. The project manager will have a face-to face meeting with the PIs every other week.

The role of the Data Monitoring Committee is to review safety and efficacy data and to make recommendations to the Trial Steering Committee. It will be comprised of four fully independent members: one chairman, one senior biostatistician, one cardiologist, and one *Baduanjin* expert from the GPHCM. The Data Monitoring Committee will meet once, prior to the start of patient recruitment, and at least once a year for those years in which patients are involved in exercise sessions.

The responsibilities of the Trial Steering Committee are to approve the main study protocol and any amendments, to monitor and supervise the trial, steering it towards its specific and overall objectives, to review relevant information from other sources, to approve and comment on project deliverables, to consider the recommendations of the Data Monitoring Committee, and to resolve any problems brought up by the Trial Management Group. It will be comprised of one independent chairman, Dr. Weihui Lu, Professor Zehuai Wen, Dr. Gaetano Marrone, Prof. Cecilia Stålsby-Lundborg, and four other independent members including at least one patient and a public involvement representative. Responsibility for calling and organizing the Trial Steering Committee meetings lies with the PIs. The Trial Steering Committee will meet at least annually, and more frequently as needed. Representatives of the Data Monitoring Committee are to be invited to all Trial Steering Committee meetings.

Before recruitment, the whole research team, including investigators, research assistants, and research nurses will be required to attend a training workshop. This will be done before the trial to ensure their strict adherence to the study protocol and their familiarity with the trial administration process. The data collected in this trial will be comprised of information recorded in CRFs, information on questionnaires, exercise logs, and accelerometers. Data will be entered using the double-entry method. Data quality is to be checked regularly by research assistants and overseen by monitors. Data monitoring will be conducted regularly with standard operation procedures by a team from the GPHCM Key Unit of Methodology in Clinical Research. Inspections will be performed regularly by the GPHCM Department of Science Research. All modifications are to be marked on the CRFs. Data managers will then recheck the data before logging it, and promptly notify the research team if any discrepancies are found. The database will be locked after all data has been cleaned. If participants withdraw from the trial during the study period, the reasons must be documented (if participants are willing) and the rate is to be statistically analyzed.

## Discussion

The EBCR has been recommended in the guidelines for CHF, but it is still in its infancy in China where EBCR services are rare in most regions. The delivery of EBCR should adopt an evidence-based approach, as well as be culturally appropriate and sensitive to individual needs and preferences. The BESMILE-HF study will establish feasibility and provide preliminary evidence on the efficacy and safety of an EBCR program, which will apply *Baduanjin* as the core component in improving exercise capacity and quality of life in patients with CHF. In particular, the BESMILE-HF program has the potential to reach the subgroup of the CHF population with limited physical functional capacity and for whom more intense physical activity is not appropriate.

A limited number of studies have emphasized the importance of individualizing exercise interventions. However, patients’ experience in CHF exercise interventions have not previously been investigated in the Chinese population. This knowledge would help clinicians and researchers in China to gain a context-specific understanding of adherence issues and how standardized interventions could be adjusted to fit individual needs, capacities, and circumstances. Therefore, the BESMILE-HF study will also explore the experience from participants’ perspectives using qualitative research.

Methodological challenges for this study will arise from evaluating a complex intervention as part of EBCR such as (1) training of the whole research team, (2) issues pertaining to the exercise records, and (3) additional physical activity monitoring. Furthermore, due to the nature of intervention, blinding is impossible in this trial. We will make every effort to ensure that outcome assessors, laboratory technicians, data managers, and statisticians are unaware of the treatment allocations.

Altogether, the results of the BESMILE-HF study might provide evidence for how to effectively deliver a contextually adapted EBCR program in China. While this study will lead to conclusions specific to the content, the findings are likely to be relevant for settings with similar lifestyles and demographic profiles, especially those with limited resources.

## Trial status

The pilot study is on-going and its recruitment began in August 2017. Data collection will finish in January 2018. Two main parts, including the RCT and the qualitative component of the BESMILE-HF study will commence in 2018.

## Additional files

Additional file 1: SPIRIT 2013 Checklist of the BESMILE-HF study. (DOCX 51 kb)

Additional file 2: Details and procedures of the BESMILE-HF program. (DOCX 30 kb)

## Abbreviations

6MWT: 6-min walking test
AEs: Adverse events
BESMILE-HF: Baduanjin Eight-Silken-Movements wIth SeLf-Efficacy building for Heart Failure
CHF: Chronic heart failure
CPX: Cardiopulmonary exercise test
CRFs: Case Report Forms
EBCR: Exercise-based cardiac rehabilitation
ECG: Electrocardiograph
GPHCM: Guangdong Provincial Hospital of Chinese Medicine
GRC: Global Rating of Change
HADS: Hospital Anxiety and Depression Scale
hsCRP: High-sensitivity C-reactive protein
IPAQ-SC: International Physical Activity Questionnaire short-form Chinese version
MACEs: Major adverse cardiac events
MLHFQ: Minnesota Living with Heart Failure Questionnaire
NT-proBNP: N-terminal B-type natriuretic peptide
PFSDQ-M: Pulmonary Functional Status and Dyspnea Questionnaire- Modified
PIs: Principle investigators
RCTs: Randomized controlled trials
SEE-C: Self-Efficacy for Exercise-Chinese
TUGT: Timed up-and-go test; VO_2_, Oxygen consumption
