# Supplementary material for: Effect of an exercise-based cardiac rehabilitation program “Baduanjin Eight-Silken-Movements with self-efficacy building” for heart failure (BESMILE-HF study): study protocol for a randomized controlled trial
Source: Trials. 2018 Mar 1;19:150. doi: 10.1186/s13063-018-2531-9 (PMC5831846; doi:10.1186/s13063-018-2531-9)
Supplement: Supplementary file 2 — Details and procedures of the BESMILE-HF program. (DOCX 30 kb) [file 13063_2018_2531_MOESM2_ESM.docx]

Additional file 2 Details and procedures of the BESMILE-HF program

| **Week** | **Activities** | **Details** | **Adherence Strategies** |
| --- | --- | --- | --- |
| -2  ~  0 | - Baduanjin learning classes - Education courses   (-2w to -1w) | Baduanjin learning class: a) we will employ two professional coaches who have engaged in teaching Baduanjin for over five years to teach and guide participants’ training; b) participants are required to learn Baduanjin until they master it, which will be confirmed by the professional coaches.  Participants will be required to attend educational courses which cover the following topics: a) chronic heart failure and exercise-based cardiac rehabilitation, and b) how to exercise during cardiac rehabilitation and in daily life. | 1. Initial demonstration of exercise technique by coaches in class (role modeling) 2. A Baduanjin picture-based educational brochure will be available (role modeling) 3. Essential instructions and feedback from coaches in class (positive feedback) 4. Evaluation of Baduanjin performance (positive feedback) 5. Exercise can be delivered in a sitting- form or a standing- form (performance accomplishment) 6. Exercise goal setting with cardiac nurses or cardiologists by phone, weekly (performance accomplishment) 7. Review of goal accomplishment and encouragement statements on progress from cardiac nurses by phone, weekly (positive feedback) 8. Patient records participation in the exercise log (performance accomplishment) 9. Patient documents concomitant medication, and adverse events in the exercise log (recognition of signs and problem-solving). |
|  | - Evaluation I - Consultation I   (-1w to 0w) | An initial evaluation of exercise capacity will be conducted by cardiologists and physiotherapists who will review medical history, cardiopulmonary exercise test results, and a Baduanjin performance by the patient.  An initial consultation lasting 20–30 minutes will be conducted by cardiologists and nurses, and will work collaboratively with patients to produce an exercise prescription guided by the initial evaluation and pre-defined SOPs. |  |
| 1  ~  4 | - Baduanjin at home - Coach-guided classes | Baduanjin at home: a) patients will be encouraged to do Baduanjin at home with the instruction of a picture-based brochure; b) Alternatively, for those who would prefer to do Baduanjin in classroom setting, participants will be able to attend a coach-guided class held at the rehabilitation center.  Three evaluations at weeks 4, 8, and 12: a) re-assessment of exercise capacity and performance of Baduanjin; b) clinical conditions and relevant physical examinations will be conducted  Three consultations at weeks 4, 8, and 12: a) difficulties in goal accomplishment and revision of exercise prescription; b) medication management, recognition and problem-solving of symptoms and signs during exercise.  Patients will be required to attend educational courses at week 12 week covering the following topics: a) long-term maintenance of exercise; and b) long-term maintenance of exercise-based cardiac rehabilitation. |  |
|  | - Evaluation II - Consultation II |  |  |
| 5  ~  8 | - Baduanjin at home - Coach-guided classes |  |  |
|  | - Evaluation III - Consultation III |  |  |
| 9  ~  12 | - Baduanjin at home - Coach-guided classes |  |  |
|  | - Evaluation IV - Consultation IV - Education courses |  |  |
